# Association between electronic cigarette use and cardiovascular disease among a United States representative population

**DOI:** 10.1016/j.ijcrp.2025.200401

**Published:** 2025-04-03

**Authors:** Humza Naqvi, Charles Searles

**Affiliations:** a Alabama College of Osteopathic Medicine, Dothan, AL, USA; b Emory University School of Medicine, Atlanta, GA, USA; c Joseph Maxwell Cleland Atlanta VA Medical Center, Decatur, GA, USA

**Keywords:** Angina, Myocardial infarction, Cardiovascular disease, NHANES, electronic cigarette

## Abstract

Electronic cigarettes have become increasingly popular, and many people use them as a safer alternative to conventional cigarettes. However, the impact of electronic cigarette use on cardiovascular health is uncertain. Here, we queried national survey data to examine the relationship between electronic cigarette use and self-reported cardiovascular disease, and we identified an association between electronic cigarette use and angina and MI but not stroke or CHF.

## 1 Introduction

Electronic cigarettes are devices that vaporize a solution containing nicotine mixed with different liquid flavors [1]. Over the last several years electronic cigarettes (e-cigarettes) have become widely available in the United States (US). Based on 2021 data, approximately 4.5 % of US adults reported using electronic cigarettes [2]. Use of e-cigarettes has been increasing over recent years, especially among young adults [3]. In comparison, 11.5 % of US adults reported conventional cigarette use in 2021 [2]. Over the past 20 years, the FDA has approved 10 different e-cigarette devices and 13 flavored tobacco e-liquids in the US [2,4]. Although e-cigarettes have been recommended as a safer alternative to conventional cigarettes, studies have indicated an association between e-cigarette use and atherosclerosis due to increased oxidative stress and endothelial dysfunction [4]. This relationship may be due to the flavoring compounds and nicotine content within e-cigarettes [4]. While multiple small scale studies have explored the potential relationship between electronic cigarette use and cardiovascular disease, results have been inconsistent [5]. Our study adds to the current research on the health risks associated with e-cigarette use by exploring the relationship between e-cigarette use and cardiovascular disease (CVD) among a large US cohort while controlling for confounding risk factors.

## 2 Methods

We assessed the relationship between electronic cigarette use and CVD among adults ages ≥20 years utilizing the National Health and Nutrition Examination Survey (NHANES) data from 2015 to 2018. NHANES is a publicly available large US survey-based dataset [6]. NHANES provides weighting variables to adjust the data to represent the US population [6]. Outcome variables for CVD included a reported history of angina, myocardial infarction (MI), congestive heart failure (CHF), and stroke. Logistic regression analyses were conducted using Stata/BE 18.5. Subpopulation models were also constructed to evaluate the relationship between electronic cigarettes and CVD by sex. Logistic regression models controlled for potential confounders, including for age, sex, race/ethnicity, tobacco smoking, diabetes, history of high blood pressure, history of high cholesterol, and BMI.

## 3 Results

Survey data from 11,189 individuals were queried. Approximately 21.6 % of subjects reported a history of e-cigarette use. Of the e-cigarette users, 1.7 % reported a history of CHF, 3.3 % reported a history of angina, 3.8 % reported a history of MI, and 2.3 % reported a history of stroke (Table 1). Among e-cigarette users with CHF, approximately 18 % reported a history of MI. E-cigarette was more common among males (55.5 %), individuals ages 20–40 years (61.9 %), and Non-Hispanic White subjects (66.8 %). After adjusting for confounding risk factors (Table 2 legend), there was a significant association between electronic cigarette use and reported history of angina (AOR 3.32, 95 % CI 1.80–6.11, p < 0.001) and electronic cigarette use and reported history of MI (AOR 2.60, 95 % CI 1.36–4.95, p = 0.005) (Table 2). There was no significant association between e-cigarette use and reported history of stroke or e-cigarette use and reported history of CHF. In addition, subpopulation regression analyses revealed that males had a significant association between e-cigarette use and angina (p = 0.04), e-cigarette use and MI (p = 0.01), and e-cigarette use and CHF (p = 0.05). Females had a significant association between e-cigarette use and angina (p < 0.001) and e-cigarette use and MI (p = 0.02), but not CHF. Moreover, using this same cohort of individuals, there was a significant association between conventional cigarette smoking and MI, stroke, angina, and CHF after controlling for the same risk factors.

**Table 1:** Demographics of subjects aged 20 years and older with and without e-cigarette usage in NHANES 2015–2018.

Characteristic	Weighted % (95 % CI)∗ E-cigaretteϕ (N = 2017)	Weighted % (95 % CI)∗ No E-cigarette ϕ (N = 9172)	P-value
**CHF**#			
Yes	1.7 (1.1–2.5)	2.5 (2.1–3.0)	**0.05**
No	98.3 (97.5–98.9)	97.5 (97.0–97.9)	
**Stroke**ψ			
Yes	2.3 (1.6–3.2)	3.1 (2.8–3.6)	**0.05**
No	97.7 (96.8–98.4)	96.9 (96.4–97.3)	
**Myocardial Infarction**σ			
Yes	3.8 (2.7–5.4)	3.3 (2.7–4.0)	0.5
No	96.2 (94.6–97.3)	96.7 (96.0–97.3)	
**Angina**ϒ			
Yes	3.3 (2.3–4.7)	2.0 (1.6–2.6)	**0.05**
No	96.7 (95.3–97.7)	98.0 (97.4–98.4)	
**Sex**			
Male	55.5 (52.8–58.1)	46.0 (45.0–47.0)	**<0.0001**
Female	44.5 (41.9–47.2)	54.0 (53.0–55.0)	
**Race/Ethnicity**			
Non-Hispanic White	66.8 (62.6–70.8)	62.1 (56.8–67.1)	**0.01**
Non-Hispanic Black	10.8 (8.5–13.6)	11.6 (9.0–14.9)	
Hispanic	13.6 (11.2–16.4)	16.0 (12.7–20.0)	
Other	8.9 (7.4–10.6)	10.3 (8.4–12.6)	
**Age**			
20–40 years	61.9 (57.9–65.9)	31.3 (29.6–33.0)	**<0.0001**
41–60 years	29.6 (25.5–34.0)	37.0 (35.1–39.0)	
61+ years	8.5 (6.6–10.9)	31.7 (29.5–33.9)	
**BMI**			
<18.5 kg/m^2^	2.4 (1.8–3.2)	1.2 (0.9–1.6)	**0.0008**
18.5–24.9 kg/m^2^	26.5 (23.8–29.4)	25.2 (23.1–27.3)	
25–29.9 kg/m^2^	26.6 (23.9–29.5)	32.4 (30.9–33.9)	
30 kg/m^2^	44.4 (40.2–48.8)	41.3 (38.8–43.7)	

Values that are statistically significant (two-side P-value ≤0.05) are in bold.

BMI, Body Mass Index; CHF, Congestive Heart Failure; CI, Confidence Interval; NHANES, National Health and Nutrition Examination Survey; E-cigarette, electronic cigarette.

∗ Weighted percentage was calculated using NHANES survey design parameters.

ϕ E-cigarette status was assessed by the question “Have you ever used an e-cigarette even one time?”

# Congestive heart failure status was assessed by the question “Has a doctor or other health professional ever told you that you had congestive heart failure?”

ψ Stroke status was assessed by the question “Has a doctor or other health professional ever told you that you had a stroke?”

σ Myocardial infarction status was assessed by the question “Has a doctor or other health professional ever told you that you had a heart attack (also called myocardial infarction)?”

ϒ Angina status was assessed by the question “Has a doctor or other health professional ever told you that you had angina, also called angina pectoris?”

**Table 2:** Association between e-cigarette usage^†^ and cardiovascular disease among US adults (aged ≥20 years) in NHANES 2015–2018.

	OR (95 % CI)		P-value	
Variable	Unadjusted	Adjusted∗	Unadjusted	Adjusted∗
*All participants*				
Angina	1.63 (1.00–2.66)	3.32 (1.80–6.11)	0.05	**<0.001**
Stroke	0.72 (0.51–1.01)	1.22 (0.66–2.28)	0.05	0.5
MI	1.17 (0.75–1.82)	2.60 (1.36–4.95)	0.5	**0.005**
CHF	0.65 (0.42–1.00)	1.43 (0.92–2.24)	0.05	0.1
*Males*				
Angina	1.08 (0.57–2.07)	2.72 (1.07–6.95)	0.8	**0.04**
Stroke	0.63 (0.43–0.90)	1.07 (0.55–2.07)	**0.01**	0.8
MI	0.77 (0.49–1.23)	2.33 (1.21–4.51)	0.3	**0.01**
CHF	0.64 (0.40–1.03)	1.80 (1.01–3.19)	0.07	**0.05**
*Females*				
Angina	2.55 (1.48–4.40)	5.07 (2.52–10.19)	**0.001**	**<0.001**
Stroke	0.84 (0.46–1.53)	1.41 (0.49–4.08)	0.6	0.5
MI	2.01 (1.08–3.74)	3.32 (1.24–8.90)	**0.03**	**0.02**
CHF	0.60 (0.24–1.50)	0.91 (0.30–2.70)	0.3	0.9

Values that are statistically significant (two-side P-value ≤0.05) are in bold.

MI, Myocardial Infarction; BMI, Body Mass Index; CHF, Congestive Heart Failure; CI, Confidence Interval; NHANES, National Health and Nutrition Examination Survey; OR, Odds Ratio; E-cig, Electronic cigarette.

∗ Logistic regression models were adjusted for age, sex, race/ethnicity, tobacco smoking, diabetes, history of high blood pressure, history of high cholesterol, and BMI.

## 4 Discussion

These findings reveal that, after controlling for confounding risk factors, e-cigarettes have a significant association with reported history of angina and MI but not stroke or CHF. E-cigarettes are also more commonly used by younger white individuals. Our findings confirm previous findings that identified a significant association between e-cigarette use and angina and MI, but no significant relationship between e-cigarette use and stroke [7]. In addition, our study adds to prior research by analyzing a contemporary, representative US population while controlling for many CVD risk factors, including conventional cigarette use. The association between e-cigarette use and angina/MI remained significant after controlling for conventional cigarette use and multiple CVD risk factors, indicating that e-cigarette use may be an independent risk factor for CVD. E-cigarettes contain various toxic compounds that have been known to cause harmful health effects. The harmful compounds, specifically formaldehyde and acrolein, in e-cigarettes can increase levels of oxidative stress, resulting in endothelial dysfunction and decreased arterial compliance. These changes may ultimately lead to CVD, including MI and angina [7]. Limitations of our study include the lack of ICD codes, under or over representation of diagnoses due to self-reported data, and inability to evaluate associations based on frequency/duration of e-cigarette use. Future studies are needed to confirm this relationship and evaluate the underlying mechanism for e-cigarette use and cardiovascular disease among a large population. Understanding the harmful effects of e-cigarettes is crucial as e-cigarette usage has been on the rise and may be an important risk factor for CVD.

## CRediT authorship contribution statement

**Humza Naqvi:** Formal analysis, Writing – original draft, Writing – review & editing. **Charles Searles:** Supervision, Writing – review & editing.

## Disclosures

None.

## Sources of funding

None.
